# An automatic spike sorting algorithm based on adaptive spike detection and a mixture of skew-t distributions

**DOI:** 10.1038/s41598-021-93088-w

**Published:** 2021-07-06

**Authors:** Ramin Toosi, Mohammad Ali Akhaee, Mohammad-Reza A. Dehaqani

**Affiliations:** 1 School of Electrical and Computer Engineering, College of Engineering, University of Tehran, Tehran, Iran; 2 Cognitive Systems Laboratory, Control and Intelligent Processing Center of Excellence (CIPCE), School of Electrical and Computer Engineering, College of Engineering, University of Tehran, Tehran, Iran; 3 School of Cognitive Sciences, Institute for Research in Fundamental Sciences (IPM), Tehran, Iran

**Keywords:** Computational neuroscience, Neural decoding

## Abstract

Developing high-density electrodes for recording large ensembles of neurons provides a unique opportunity for understanding the mechanism of the neuronal circuits. Nevertheless, the change of brain tissue around chronically implanted neural electrodes usually causes spike wave-shape distortion and raises the crucial issue of spike sorting with an unstable structure. The automatic spike sorting algorithms have been developed to extract spikes from these big extracellular data. However, due to the spike wave-shape instability, there have been a lack of robust spike detection procedures and clustering to overcome the spike loss problem. Here, we develop an automatic spike sorting algorithm based on adaptive spike detection and a mixture of skew-t distributions to address these distortions and instabilities. The adaptive detection procedure applies to the detected spikes, consists of multi-point alignment and statistical filtering for removing mistakenly detected spikes. The detected spikes are clustered based on the mixture of skew-t distributions to deal with non-symmetrical clusters and spike loss problems. The proposed algorithm improves the performance of the spike sorting in both terms of precision and recall, over a broad range of signal-to-noise ratios. Furthermore, the proposed algorithm has been validated on different datasets and demonstrates a general solution to precise spike sorting, in vitro and in vivo.

## Introduction

Neural activity monitoring is one of the bases of circuit-level brain science. To do so, electrophysiologists usually place an electrode in brain tissue to record the activity of neurons in an extracellular manner. The action potential or spike is produced by the electrical currents induced to flow in the extracellular space around an active neuron. The current flows in the extracellular space would be mapped to an electric potential in the electrode. The recorded potential is a combination of multiple neuron activities corrupted by noise. Hence, it is required to differentiate neuronal activities using spike sorting for analyzing the extracellular data. The spike sorting is the process of assigning each detected spike to the corresponding neurons.

Spike sorting algorithms usually consist of three main steps^1^. The first step is the detection of spiking activities, where spikes are extracted from the recorded band-passed filtered-signal. A common way to detect spikes is to determine activities that cross a threshold^1^. The threshold is usually calculated based on the estimated noise variance^2^ without considering dynamic changes of the recorded signal, which causes spike loss or false alarms. In the second step, features are extracted from spike wave-shapes. Principal component analysis (PCA)^3–7^ and wavelet decomposition^6,8,9^ are some examples of typical methods for feature extraction^2^. Finally, in the third step, a clustering method is used to assign each spike to separate units. The clustering could be completely handled manually by the human intervention, e.g., Xclust, M.A. Wilson^10^ and Offline Sorter, Plexon^11^. In an alternative scenario, results are modified after an initial automated sorting^5,12^. The modifications include merging, deleting, or splitting clusters. However, manual clustering could have more than 20% error rate^13^ and is not practical as recorded data grows up. The automatic spike sorting algorithms have been proposed to overcome these issues. Some examples of common clustering methods are mixture modeling^5,14–17^, template matching^18,19^, and density-based clustering^20^ (for a review on clustering methods see the work of Veerabhadrappa^21^). Most of the current spike sorting algorithms concentrate on clustering and parallelism to support microelectrode array^21^.

Although many automatic spike sorting methods have been introduced so far, the instability of clusters originated from the spike wave-shape distortion is poorly investigated. The change of brain tissue around chronically implanted neural electrodes usually causes spike wave-shape distortion and raises the crucial issue of spike sorting in unstable conditions. On the other hand, changing the distance between electrode and neuron results in that the wave-shape clusters in feature space not to be well-formed. These problems cause false alarms and misalignment, which raise the need for adaptive detection procedures to apply on the detected spikes and clustering methods to overcome not well-formed clusters. For these reasons, we introduce a fully automatic spike sorting algorithm which applies multi-point alignment, statistical filtering, and skew-t clustering. Multi-point alignment and statistical filtering are applied on the detected spikes to address spike wave-shape distortion. Besides, the benefit of the mixture of skew-t distributions clustering is to be able to deal with non-symmetrical clusters.

Many suggested automatic spike sorting algorithms work on the detected spikes extracted from traditional threshold-based and single-point alignment procedures^22,23^. Here, we propose an adaptive method to improve the quality of detected spikes in terms of false alarm. Both adaptive multi-point alignment and statistical filtering are independent of the spike wave-shape detection procedure and can be applied to every detected spike. Beside the correction of the spike starting time, the proposed alignment reduces the within-cluster variability and the number of principal components that are sufficient to be taken as features. Statistical filtering employs statistical characteristics of the spike wave-shapes to remove false alarms (where noises detected as spikes). As we observed great progress on the timing of spiking activity, the false alarm reduction is a significant achievement.

To overcome the skewed and non-symmetrical spike groups, a new clustering method based on the mixture of skew-t distributions is introduced. Mixture modeling is one of the successful clustering methods usually employed in spike sorting algorithms^23,24^. Although primary works have been focused on Gaussian mixture models, it has been shown that the mixture of t-distribution is more powerful than the Gaussian mixture in modeling neural data^17,24^. By using the mixture of Gaussian or t-distribution, we assume that the clusters are symmetrical, while skewed clusters are one of the challenges in the sorting algorithms^25–27^. We propose a new robust clustering method based on skew-t distribution to handle non-symmetrical clusters and preserve powerful properties of symmetrical t-distribution like being heavy tailed.

The proposed algorithm evaluated using synthesized and two real datasets: with and without ground truth information, through several experiments. Our algorithm improved the detection accuracy even in noisy environments. The proposed adaptive detection could enhance the performance of any clustering method. In fact, it is not limited to the mixture of skew-t distributions clustering. We also developed an open-source toolbox based on the proposed algorithm. The toolbox provides different visualizations and manual sorting functions alongside the automatic one to improve the results. The proposed toolbox is developed based on MATLAB and is freely available at https://github.com/ramintoosi/ROSS.

## Methods

Our proposed spike sorting algorithm includes three main parts: preprocessing, adaptive detection, and clustering. In the preprocessing, an initial detection is performed. Next, in the adaptive detection, procedures like noise removal and multi-point alignment would be handled. Finally, the spikes are clustered automatically after the feature extraction procedure using a mixture of skewed multivariate t distributions.

### Preprocessing

The raw data includes the spike activities combined with local field potentials (LFP), network sub-threshold activities, and noise. Thus, the raw signal can be explained as follows:

1 $$r(t) = l(t) + s(t) + n(t)$$

where *t* is the time, *r* is the raw signal, and *l*, *s*, and *n* are the LFP, spike activity, and noise, respectively. To reduce the effect of unwanted components a fourth-order Butterworth bandpass filter between 300 to 3000 Hz is applied. The designed filter is a causal infinite impulse response (IIR) filter. The main advantage of IIR filters over finite impulse response (FIR) ones is their efficient hardware and software implementations since they reach to certain specifications with a lower order. However, since the causal IIR filters have a non-linear phase response, they can alter the spike wave-shapes dramatically or makes noises look similar to spikes^28^. This shape altering could also be the source of distraction in discriminating the pyramidal and inhibitory neuron spikes^29^ or finding relations between intra and extra-cellular activities^30^. To tackle this problem, a zero-phase filter^31^ is implemented by filtering the signal in forward and reverse orders in an offline mode (Supplementary Appendix S1 online).

To detect spikes, first, we need to estimate the standard deviation (std) of noise, i.e., *σ*_*n*_, which is calculated in the following according to the Donoho’s rule^28^:

2 $$\sigma _{n} = \frac{{median\left( {\left| {r_{f} } \right|} \right)}}{{0.6745}}$$

where *r*_*f*_ is the filtered version of the raw signal. The spike detection threshold is *t*_*s*_ = 3×*σ*_*n*_. It should be noted that, based on the employed datasets, we only use negative thresholding, i.e., detection occurs when *r*_*f*_ < *t*_*s*_. To increase the detection accuracy and reduce false alarms, not every threshold crossing sample would be considered as a spiking activity. Given the inherent shape of an action potential, we expect the next samples to pass the threshold too. Thus, the spiking activity would be detected if

3 $$r_{f} (m_{s} + m_{i} ) < - t_{{s~~}} \forall m_{i} \in \{ 0,1, \ldots ,m_{d} \}$$

where *m*_*s*_ is the first point that crosses the threshold and *m*_*d*_ is the number of samples that must pass the threshold successively. Finally, the detected spikes would be aligned with the first local minimum after *m*_*s*_. The local minimum is the point where there exists no point with a smaller value up to one millisecond after that. Using these local minimums reduces the detection error of spike time caused by noise.

### Adaptive detection

After initial detection of the spiking activities, the samples of 0.5 milliseconds before and one millisecond after the detected point would be captured as spike samples. In the adaptive detection phase, first, it is tried to remove noise values from the detected samples which we call *statistical filtering*. Next, a novel multi-point alignment procedure would be applied on spike samples.

#### Statistical filtering

By careful analysis of noise samples that have been detected as spikes, we found two characteristics discriminating noise from the real spikes. The two characteristics are the absolute value of average and std of samples of the waveform. For falsely detected spikes, the std of samples is large and their average is far from zero. Thus, we defined our statistical filtering based on these two parameters. To filter the false alarms, spikes whose absolute value of average and std parameters are greater than a threshold are removed. The empirically calculated thresholds are set to one and three for the average and std, respectively.

Intuitively speaking, high std wave-shapes are probably the results of higher noise distortion. As we know, the higher noise power, the higher std of noise.

4 $$\sigma _{{s + n}}^{2} ~ = \sigma _{s}^{2} ~ + \sigma _{n}^{2}$$

where *s* is the spike wave-shape and *n* is noise. But noise addition does not change the average of spike wave-shape. Thus, high average spike wave-shapes are the results of abnormal spike shapes, such as extremums with high bandwidth. Finally, we note that these properties are also observed experimentally over a range of real recording sessions.

#### Multi-point alignment

**Figure 1:**
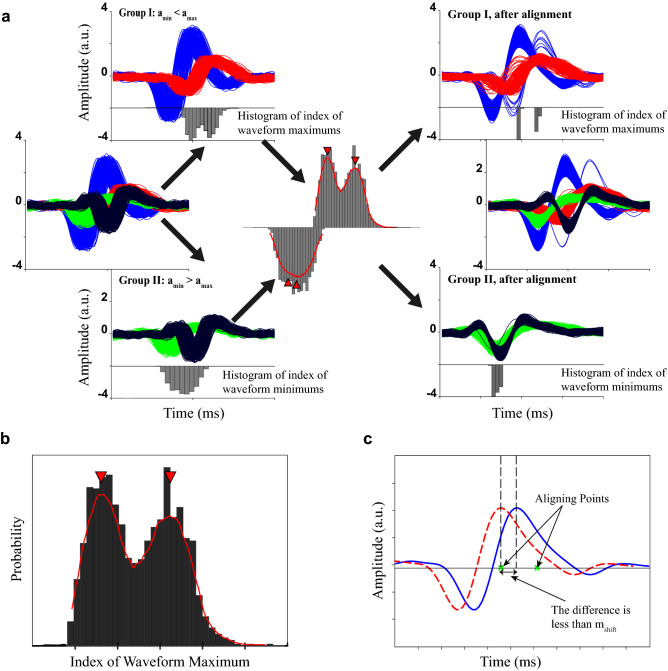
An intuitive example of the proposed multi-point alignment procedure. (**a**) Alignment of synthesized waveforms. The y-axis shows amplitude with arbitrary unit (a.u.). The synthesized waveforms are designed to form four distinct shapes. The waveforms are grouped according to the amplitude of their maximums and minimums. Then, the peak of the smoothed histogram of extremum indices is found, as the aligning points. Finally, the waveforms are aligned regarding the aligning points. (**b**) The histogram of the peak locations in waveforms. The smoothed cubic spline fitted to the histogram is shown by the red line. The locations of two local maximums are indicated using red triangles. (**c**) The blue solid line is the original waveform. The aligning points corresponding to the local maximums in (**b**) are also depicted using green crosses. If the difference between the peak location of the waveform and the nearest aligning point is less than *m*_*shift*_, the waveforms are aligned by an appropriate shift, forming the aligned waveform illustrated by dashed red line.

A multi-point aligning procedure is introduced in this section. The proposed procedure takes both the minimum and maximum value of the detected spike into account. Assume that *s*_*pi*_ is the *i*’th detected spike. First, samples are grouped based on the amplitude of their minimums or maximums, i.e., *a*_*min*_ and *a*_*max*_. Samples in which *a*_*min*_ < *a*_*max*_ are in one group and the other samples are gathered in the other one. The rest of the procedure would be applied to each group separately. Assuming the group where *a*_*min*_ < *a*_*max*_, the histogram of the time indices of the maximum values would be calculated as shown in Figure 1b. The histogram is smoothed using a cubic spline interpolation. Then, the *m*_*peak*_ greatest peaks in the histogram would be considered as aligning points (thus, *m*_*peak*_ is the number of selected peaks) (Figure 1b). For each sample in the group, if the maximum of the sample could reach the nearest aligning point by the maximum shift of *m*_*shift*_ point, it would be shifted to be aligned with the nearest aligning point (Fig. 1c) (thus, *m*_*shift*_ is the maximum allowed shift for aligning). The aligning procedure is illustrated in Figure 1a.

There exist two parameters in the alignment process, i.e., *m*_*peak*_ and *m*_*shift*_. Here, we discussed the extreme cases of these two parameters. When *m*_*peak*_ is one, the procedure seeks to align all spikes into one unique point. In this way, if the difference between two neuron spikes is the index of their extremum, then the alignment misleads the clustering process by removing a discrimination characteristic. The other extreme case is when *m*_*peak*_ is equal to the number of samples. In this scenario, the procedure does not align any waveform. Thus, increasing the value of *m*_*peak*_ reduces the aligning effect. The other parameter is the maximum allowed shift for aligning a sample, i.e., *m*_*shift*_. Small values of this parameter reduce the effect of the alignment. Also, large shifts dramatically change the spike wave-shapes which is not desirable. After alignment, the tails of the spike samples, which do not carry important information about the spike wave-shape would be eliminated.

### Feature extraction

Applying PCA, the spike samples are projected into a low dimensional space in the feature extraction step. Instead of using a fixed number of components, the number of features selected for clustering is determined based on the amount of the variance preserved by the principal components. Assume *λ*_*i*_ is the *i*’th greatest principal component variance. We select *n*_*c*_ components where *n*_*c*_ is the smallest solution that satisfies the following condition:

5 $$\frac{{\mathop \sum \nolimits_{{i = 1}}^{{n_{c} }} \lambda _{i} }}{{\mathop \sum \nolimits_{{i = 1}}^{N} \lambda _{i} }} \ge ~0.95$$

where *N* is the total number of components. Intuitively, the new low dimensional space preserves 95% of the variance in the original high dimensional space to describe spike wave-shapes. This procedure allowed the algorithm to adaptively choose the dimension of the feature space based on the spike wave-shape variations. However, choosing a large number of components may affect the clustering process because of the *curse of dimensionality*^32^. Therefore, if the number of selected components by (5) exceeds a predefined value, *N*_*pca*−*max*_, the algorithm chooses the first *N*_*pca*−*max*_. Throughout this paper, we fixed *N*_*pca*−*max*_ = 15.

### Automatic spike sorting using mixture of skew-t distribution

In this section, we propose a new automated spike clustering algorithm based on mixtures of skew-t distribution. The expectation maximization (EM) algorithm applied in this section is based on the work of Cabral et al.^33^. In their work, the skew normal independent (SNI) distribution family is discussed. The proposed clustering method is based on the skew-t distribution which is a member of SNI family^33^. We run the EM algorithm to fit the mixture of skew-t distributions for data/spike samples.

To define the multivariate skew-t (ST) random variable statistics, first we need to define the multivariate skew-normal (SN) one. A p-dimensional random variable X follows the SN distribution, if its distribution is given by:

6 $$SN_{p} \left( {x|\mu ,\Sigma ,\lambda } \right) = ~2\phi _{p} \left( {x|\mu ,\Sigma } \right) \times \Phi \left( {\lambda ^{T} \Sigma ^{{ - \frac{1}{2}}} \left( {x - \mu } \right)} \right)$$

where *µ* is the *p* × 1 location vector, Σ is *p* × *p* positive definite dispersion matrix, and *λ* is the *p* × 1 skewness parameter vector. *φ*_*p*_(.|*µ,*Σ) is the probability density function (PDF) of the p-dimensional normal random variable, and $\Phi$(*.*) stands for the standard univariate normal cumulative distribution function (CDF).

Assume the following random variable, *Y*,

7 $$Y~ = ~\mu + ~U^{{ - \frac{1}{2}}} Z$$

where *µ* is a p-dimensional location vector, *Z* ∼ *SN*(0*,*Σ*,λ*), and *U* is a positive random variable, independent of *Z*, with PDF h(.|v). If *U* follows a $Gamma\left( {\alpha ~ = \frac{v}{2},\beta = \frac{v}{2}} \right)~$ distribution, i.e.

8 $$h\left( {u|v} \right) = ~Gamma\left( {\alpha ~ = \frac{v}{2},\beta = \frac{v}{2}} \right) = \frac{{\left( {\frac{v}{2}} \right)^{{\frac{v}{2}}} }}{{\Gamma \left( {\frac{v}{2}} \right)}}u^{{\frac{v}{2} - 1}} e^{-{\frac{v}{2}u}}$$

where Γ(*.*) is the Gamma function, then, *Y* follows a multivariate ST distribution with the location vector *µ*, dispersion matrix Σ, skewness vector *λ*, and *v* degrees of freedom. The distribution of *Y*, could be simplified based on (6)–(8) as follows:

9 $$ST_{p} (y|\mu ,\Sigma ,\lambda ,v)~ = ~2t_{p} (y|\mu ,\Sigma ,v)~ \times T\left( {\sqrt {\frac{{v + p}}{{v~ + ~d_{\Sigma } \left( {y,\mu } \right)}}} \lambda ^{T} \Sigma ^{{ - \frac{1}{2}}} \left( {y - \mu } \right)|v + p} \right)$$

where *t*_*p*_(*.*|*µ,*Σ*,v*) denotes the pdf of the p-dimensional student-t distribution with the mean *µ*, dispersion matrix Σ, and *v* degrees of freedom. Also, *T*(*.*|*v*+*p*) is the standard univariate student-t CDF with *v*+*p* degrees of freedom. *d*_Σ_(*y,µ*) = (*y*−*µ*)^*T*^Σ^−1^(*y*−*µ*) is the Mahalanobis distance between *y* and *µ*.

#### Mixture of multivariate skew-t distribution

In the mixture models, we assume that each spike sample, denoted as *s*_*i*_, originates from a finite set of known distributions (or components) with unknown parameters. First, it is supposed that the number of distributions (components or neurons), *g*, is known. In the next subsection, when the mixture model is used for clustering, *g* would be determined automatically. Assuming the spikes are *i.i.d* random variables denoted by *S*, the likelihood function can be written as:

10 $$p\left( {S|\Theta } \right) = \mathop \prod \limits_{{i = 1}}^{N} p\left( {s_{i} |\Theta } \right) = \mathop \prod \limits_{{i = 1}}^{N} \mathop \sum \limits_{{j = 1}}^{g} \uppi _{j} p\left( {s_{i} |\Theta _{j} } \right),\quad \sum \uppi _{j} = 1$$

where *N* is the number of total samples (spike wave-shapes), *Θ*_*j*_ is the distribution parameter set, and *π*_*j*_ is the mixing probability. In the proposed method, components are ST distributions (i.e. *p* = *ST*_*p*_) with the parameter set *Θ*_*j*_ = {*µ*_*j*_*,*Σ_*j*_*,λ*_*j*_*,v*}. To find the optimum set of parameters, we employ the EM algorithm. The details of the EM formulation are provided in Supplementary Appendix S2 online.

To find the optimum parameters, the algorithm iteratively does the expectation and maximization steps respectively, until a stop criterion is met. Intuitively, the algorithm should stop when the parameters reach a stable value in the updating procedure. Mathematically, the algorithm stops, if the following condition is met:

11 $$\mathop {\max }\limits_{{j,a_{j} \in \Theta _{j} }} \left| {\left| {a_{j}^{{\left( {k + 1} \right)}} - a_{j}^{{\left( k \right)}} } \right|} \right| < l_{{EM}} .$$

The condition captures the maximum change among all parameters of all components, and then checks if it is smaller than a predefined threshold, i.e., *l*_*EM*_. The EM algorithm is summarized in Algorithm 1, where *Θ*_*init*_ is the initial values for the parameters.
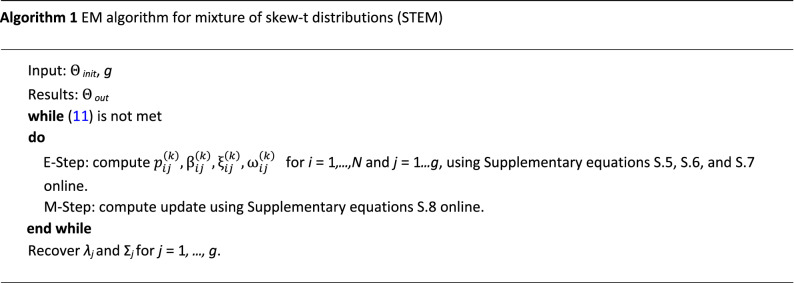


#### Clustering method

In the EM algorithm, it is assumed that the number of components, i.e., *g* is known. But in the automatic spike sorting problem, the number of components or neurons is unknown and the algorithm has to determine it. As described in Algorithm 1, the output of the EM algorithm is defined as *Θ*_*out*_ = *STEM*(*Θ*_*init*_*,g*), where *Θ*_*init*_ and *Θ*_*out*_ are the initial and output (optimum) parameters as shown in Algorithm 1. The clustering method searches the best value of *g* within the interval [*g*_*min*_*,g*_*max*_]. A simple way is to run STEM for *g* = *g*_*min*_*, ..., g*_*max*_ independently. Besides, the random initialization of parameters could be used for each run. Then, a criterion determines the optimum value of *g*. Here, to speed up the algorithm, we exploit the output of the previous run of the STEM to initialize the parameters of the next run.

The proposed clustering method starts from *g* = *g*_*max*_ and continues the search toward *g* = *g*_*min*_. In the first run, when *g* = *g*_*max*_, we use the fuzzy c-means clustering (FCM) algorithm to calculate *Θ* instead of using random initialization. The FCM method outputs the cluster centers and a fuzzy partition matrix, *U*, where *U*_*ij*_ is the probability that the *i*’th spike belongs to the *j*’th cluster. *µ* is initialized by the centers of the FCM results. The dispersion matrix is

12 $$\Sigma _{j} = \frac{{\mathop \sum \nolimits_{{i = 1}}^{N} U_{{ij}} \left( {s_{i} - \upmu _{j} } \right)\left( {s_{i} - \upmu _{j} } \right)^{T} }}{{\mathop \sum \nolimits_{{i = 1}}^{N} U_{{ij}}}}.$$

Also, the initial value of the skewness vector, *λ* is,

13 $$\lambda = sign\left( {\mathop \sum \limits_{{i = 1}}^{N} U_{{ij}} \left( {s_{i} - \upmu _{j} } \right)^{{. \wedge }} 3} \right)$$

where *.*∧ denotes the element wise power.

After running the STEM algorithm, the estimated parameters would be exploited to initialize the next run. Suppose that *Θ*_*out,k*_ is the estimated mixture parameters in the *k*’th step. The initial values for the next run would be calculated as follows:

- Find the component with minimum mixing probability, *m* = argmin_*l*_* π*_*l*_
- Remove *π*_*m*_ and normalize the remaining mixing probabilities such that, ∑_*l*_* π*_*l*_ = 1.
- Remove *µ*_*m*_, Σ_*m*_, and *λ*_*m*_.
- Use the output degree of freedom, *v*, to initialize the next one.
- Set *g* ← *g* − 1.
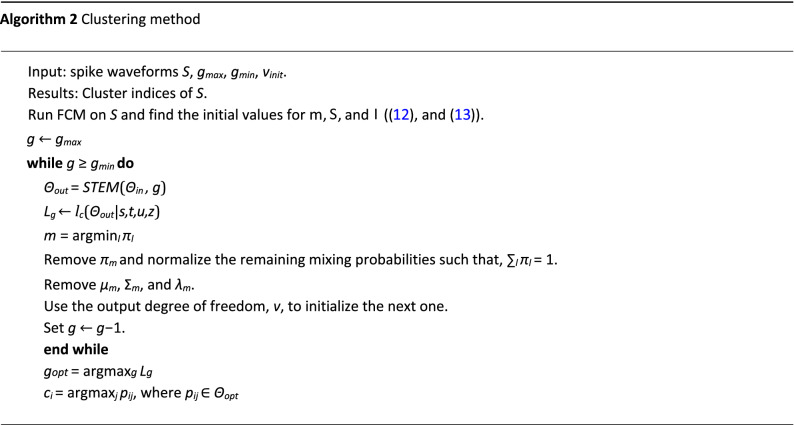

In order to find the best value of the number of components, *g*, we use the likelihood function in Supplementary equation S.3 Online. Assume *L*_*g*_ denotes *L*_*c*_(*Θ*|*s,t,u,z*) when the number of components is *g*, then the optimum value of *g*, i.e., *g*_*opt*_ is,

14 $$g_{{opt}} = \arg \mathop {\max }\limits_{g} L_{g} .$$

Also, one can terminate the algorithm if increasing of *L*_*g*_ is observed. Finally, the cluster index of each spike would be obtained as follows:

15 $$c_{i} = \arg \mathop {\max }\limits_{j} p_{{ij}}$$

where *c*_*i*_ is the cluster index of *s*_*i*_. The proposed clustering method is summarized in Algorithm 2.

### Spike sorting index

Each fully automated spike sorting algorithm could be evaluated in terms of both detection and clustering results. To the best of our knowledge, there exist few efforts to introduce a metric to evaluate the whole process of spike sorting considering both detection and clustering results^34^. Instead, various metrics have been developed to capture the performance of the detection and clustering phases separately. Therefore, here we use normalized mutual information to introduce spike sorting index (SSI) that captures the total performance by integrating both detection and clustering performances. There exist three sources of performance degradation in the spike sorting algorithms: (i) miss detection, (ii) false alarm, and (iii) clustering error. To calculate SSI, a new and unique ground truth cluster is assumed for the falsely detected spikes. In other words, since we have no ground truth for false alarms, we assume that they all belong to a hypothetical cluster. Then, missed spikes are randomly distributed among the existing predicted clusters. Finally, SSI is defined as the normalized mutual information between true and predicted cluster indexes as follows^23^.

16 $$SSI = \frac{{I\left( {C,C^{\prime } } \right)}}{{\max \left( {I\left( {C,C} \right),I\left( {C^{\prime } ,C^{\prime } } \right)} \right)}}$$

where *C* and *C*′ are the true and predicted cluster indexes, and *I*(*.,.*) is the mutual information function. Any increment in the miss detection, false alarm, or clustering error, results in decrement in SSI. The effect of each kind of error on the proposed SSI has been investigated in Supplementary Appendix S4 Online.

## Results

Here, the performance of the proposed algorithm is investigated using three different datasets, including one simulated and two real datasets. First, the details of the datasets are described. Then, we go through the details of the experiments and the results are discussed. Through this section, the proposed algorithm is compared with various spike sorting algorithms. Also, the advantages of different contributions are investigated by comparing them to Shoham’s algorithm^17^. In this algorithm, they used a common procedure of spike detection and alignment which is bandpass filtering, thresholding, and aligning based on the wave-shape extremums, such as the one used by WaveClus^9^. From now on, the algorithm of Shoham^17^ and the proposed algorithm is referred to as MTD (mixture of t-distributions), and MSTD (mixture of skew-t distributions). In the following experiments, all statistical comparisons are calculated using the signed-rank test.

To evaluate the detection performance, the *precision* and *recall* metrics are calculated. A detected spike is considered as a true positive if there exists at least one spike in its one-millisecond vicinity in the ground truth. The precision metric measures the ratio of truly detected spike and could be calculated using the following formula:

17 $$Precision = \frac{{{\text{true}}\;{\text{positive}}}}{{{\text{true}}\;{\text{positive}} + {\text{false}}\;{\text{positive}}}}.$$

On the other hand, the recall metric measures the ratio of the spikes detected by the detection procedure. It can be stated as follows:

18 $$Recall = \frac{{{\text{true}}\;{\text{positive}}}}{{{\text{true}}\;{\text{positive}} + {\text{false}}\;{\text{negative}}}}.$$

The high precision means fewer false positives (false alarms) and high recall means fewer false negative (miss) spikes. To evaluate the clustering quality and also considering the ground truth, the accuracy and purity metrics are employed. For accuracy, the label of each cluster is determined by the first dominant neuron in that cluster which has not been already assigned. The dominant neuron in each cluster is the neuron that has the greatest number of spikes in that cluster. Accuracy could be calculated using the following formula.

19 $$Accurracy = \frac{{{\text{Number}}\;{\text{of}}\;{\text{correctly}}\;{\text{classified}}\;{\text{spikes}}}}{{{\text{Total}}\;{\text{number}}\;{\text{of}}\;{\text{spikes}}}}$$

For purity, the percent of the number of dominant neuron samples in a cluster is considered as the purity of that cluster. The average purity of all clusters is calculated as the clustering purity. The purity could be calculated as follows.

20 $$purity = \frac{1}{N}\mathop \sum \limits_{{c \in \mathcal{C}}} \mathop {\max }\limits_{{d \in \mathcal{D}}} \left| {c \cap d} \right|$$

where $\mathcal{C}$ and $\mathcal{D}$ are the sets of detected and true clusters and *N* is the total number of spikes.

### Datasets

A synthetic dataset is used to show the correctness and efficiency of the proposed algorithm. To simulate neural recording data, the noisy spike generator toolbox provided by Smith and Mtetwa^35^ is employed^36^. Using this tool, 200 sessions of recording are simulated. Each session contains 200 seconds of recording with a sampling rate of 40KHz. In each session, there exist four neurons with Poisson distribution and a refractory period of one millisecond. The other options are left as their defaults. This resulted in approximately 30000 spikes in each session.

For the first real dataset, we employed the simultaneous intracellular and extracellular recordings from hippocampus region CA1 of anesthetized rats, provided by Henze et al.^30,37^. Sessions with appropriate intracellular recording were selected manually. We consider the intracellular data as the ground truth to evaluate the performance of the proposed spike detection and preprocessing. The third dataset is a recording of a macaque. These sessions are collected from the inferotemporal cortex area, while the monkey does a rapid serial visual presentation task. It consists of 200 sessions with sampling rate of 40 kHz.

### MSTD methodology overview

**Figure 2:**
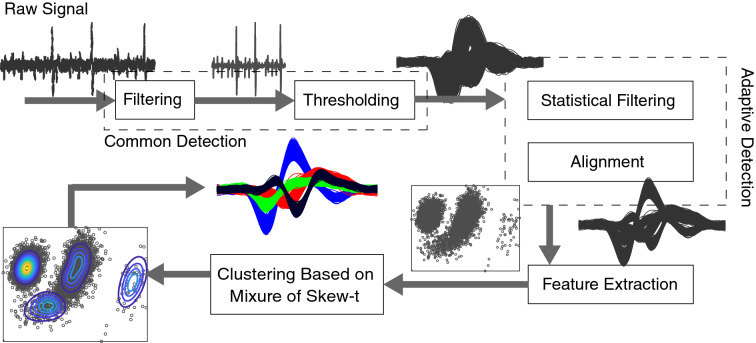
Illustration of the procedure of the proposed automatic spike sorting algorithm. First, the raw signal is fed into a common detection procedure. As an example, first, the signal is filtered, then, an initial detection is performed by applying a simple thresholding procedure. Thereafter, the proposed adaptive detection procedure takes the initially detected spikes and performs a set of procedures to improve the detection results and make them ready for feature extraction. In adaptive detection, first, the noise which is detected as waveforms are removed using the proposed statistical filtering. Then the proposed multi-point alignment seeks to align the waveforms based on the histogram of the extremums. Then, an adaptive number of principal components are considered as features. Finally, the samples are fed to the clustering method based on the mixture of skew-t distributions.

Figure 2, graphically illustrates the procedure of the proposed algorithm. In the preprocessing step, first, the signal is bandpass filtered using the zero-phase filtering method. Next, using simple thresholding, initial detection of spikes is performed. The threshold is calculated as three times of the standard deviation of the noise. At this point, a bunch of detected waveforms is ready for the proposed adaptive detection procedure. Here, statistical filtering runs to reduce the system false alarm by removing noises that are mistakenly detected as a spike. For this sake, the waveforms that their averages or standard deviations exceed a predefined value would be removed from data. Then, the proposed multi-point alignment procedure seeks to align the waveforms based on the histogram of their extremums. The main point of our multi-point alignment is to use different points to align waveforms instead of only considering its minimum or maximum. The next step is feature extraction, where we adaptively choose some principal components as features. Finally, the samples are fed to the proposed clustering method based on the mixture of skew-t distributions. The proposed clustering method estimates the parameters for skewed distributions and automatically finds the best number of neurons.

### Comparison of MSTD with state-of-the-art sorting algorithms

**Figure 3:**
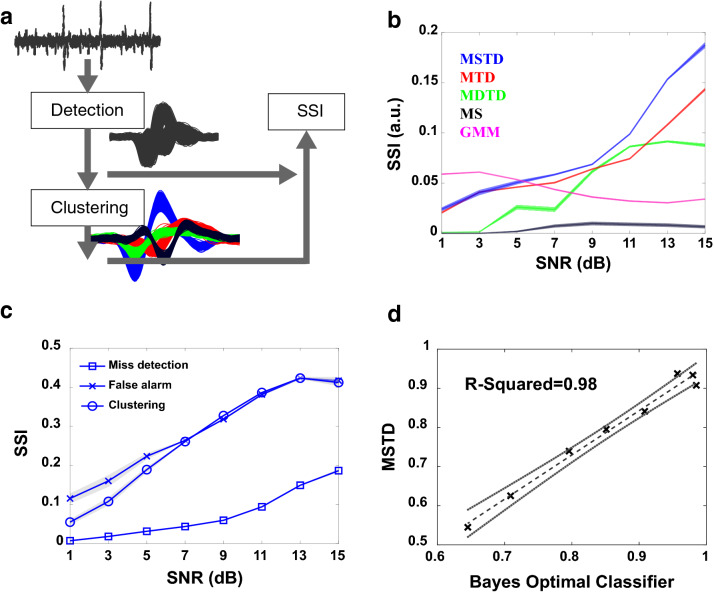
Comparison of the MSTD performance with four state-of-the-art spike sorting algorithms. Results are obtained using the synthesized dataset. (**a**) Illustration of the SSI metric. It considers both detection and clustering outputs integrated into a single value. (**b**) Comparison of the proposed fully automatic spike sorting algorithm with four state-of-the-art algorithms employing SSI. The shaded area is the standard error. The proposed MSTD algorithm outperforms all other compared algorithms considering both detection and clustering performances, simultaneously. Only for SNRs lower than 5 dB, GMM has a better performance than the proposed algorithm. (**c**) The effect of miss detection, false alarm, and clustering performance on SSI. The MSTD algorithm has the lowest performance in terms of miss detection. (**d**) CA-CA plot between MSTD and the Bayes optimal classifier to investigate the robustness of the proposed method. The R-squared of the fitted line with the least square method is 0.98.

In the first experiment, the proposed spike sorting algorithm, MSTD, is compared to four state-of-the-art algorithms, including Mountainsort^22^ (MS), mixture of drifting t distributions^24^ (MDTD), Gaussian mixture based^23^ (GMM), and mixture of tdistributions^17^ (MTD), using synthesized dataset (the first dataset introduced in the Datasets section). As depicted in Figure 3a, we use the proposed SSI metric to compare the overall performance of both detection and clustering phases, simultaneously. Results are depicted in Figure 3b. As can be seen, the proposed MSTD, outperforms other state-of-the-art spike sorting algorithms for signal to noise ratios (SNR) greater than 7dB. The low performance of the MS algorithm is probably a direct result of the poor detection performance as shown in Supplementary Figure S1 Online. GMM also shows a relatively steady performance in comparison to the other algorithms, where it outperforms MSTD in SNRs lower than 7dB; however, its performance does not increase with SNR. Further investigations on detection and clustering phases of GMM method, reveals that the false alarm increases in high SNRs. This is probably because of the fact that the GMM uses signal variance to determine the threshold instead of noise variance estimation^23^. The performance of MSTD could be analyzed in terms of miss detection, false alarm, and clustering accuracy, separately, as depicted in Figure 3c. The performance in terms of false alarm and clustering is roughly the same and higher than miss detection. Therefore, MSTD tends to have more misses than false alarms, which is the result of statistical filtering. One way to increase the recall (decrease miss detection) is to apply lower thresholds in the detection phase. Finally, to investigate the robustness of the proposed method, we use classification accuracy (CA) against the Bayes optimal classifier. A CA-CA plot of MSTD and Bayes optimal classifier is depicted in Figure 3d. A linear regression with least square method is used to fit a line for CA-CA points. The slope and R-squared of the fitted line are 0.88 and 0.98 respectively, which shows the robustness of the proposed method. Note that while Figure 3d shows the robustness of the MSTD method, the same reduction in the SNR value has more effect on the SSI metric for MSTD than MTD (Figure 3b and Supplementary Figure S4 Online). In the next experiments, the performance of different parts of our proposed spike sorting algorithm is investigated separately.

### Application and validation of adaptive detection on synthesized dataset

**Figure 4:**
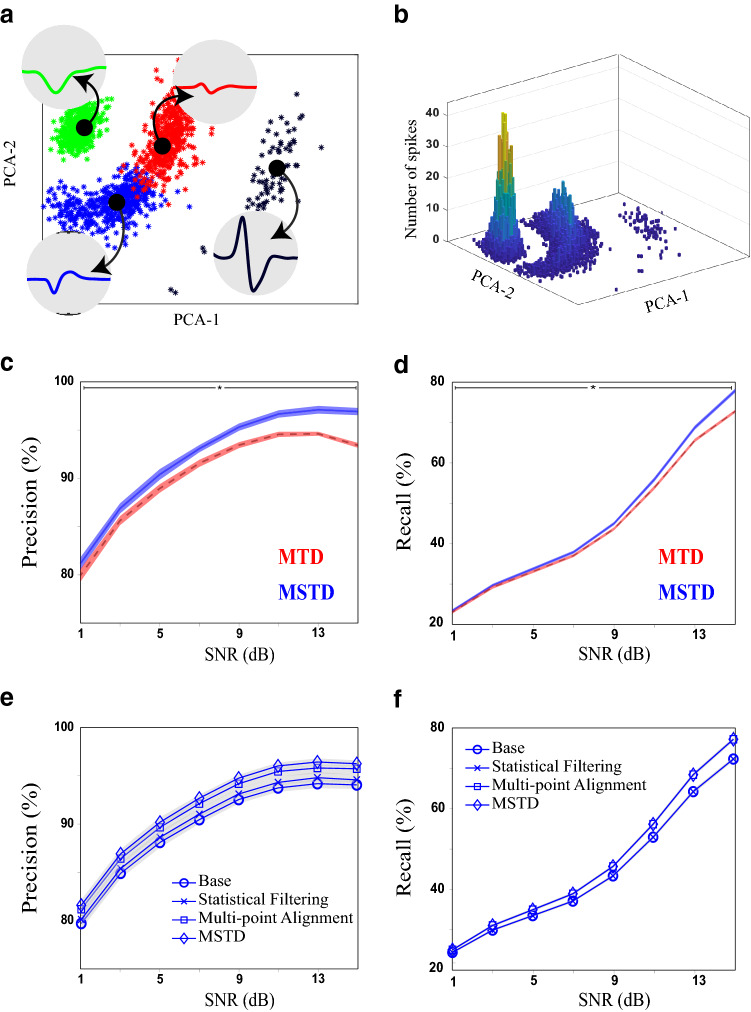
Evaluating the detection phase of the MSTD algorithm using synthesized dataset^35,36^ in the detection phase. The dataset contains 200 sessions of 200-s recording with a sampling rate of 40 kHz. (**a**) A sample session is visualized using its two first principal components. There exist four distinct neurons distinguished by four colors. The average waveform of each neuron is also indicated in a 1.2-ms interval. (**b**) A sample distribution of spike waveforms. The x and y axes are the first two principal components of waveforms and the z-axis is the frequency of spikes. (**c**, **d**) Evaluating the proposed detection (multi-point alignment, and statistical filtering procedures). The precision (**c**) and recall (**d**) of MSTD in the detection phase is compared with MTD. The shaded area is the standard deviation. The horizontal line with the star shows where the values differ significantly. (**e**, **f**) The effect of statistical filtering and multi-point alignment on detection performance in terms of precision and recall. The MSTD method without any alignment and noise removal is depicted by "Base". The effect of multi-point alignment is higher than statistical filtering. In terms of recall, since statistical filtering only deals with false alarms, it has no effect on the performance.

In the next experiment, we used the simulated dataset to evaluate the proposed detection and preprocessing procedures. The true spikes of a sample session are visualized using the first two components of PCA in Figure 4a. As can be seen, there exist four distinct groups of spikes with different colors. The distribution of spikes is also illustrated in Figure 4b. For better evaluation, the population of each group is chosen from a range of highly populated to the sparse one. In this experiment, eight Gaussian noise signals with different powers are added to each recording to produce noisy signals with a controlled SNR. Then each signal is filtered and spikes are detected as described in the adaptive detection procedure. Each noisy recording, in each SNR, is fed to the detection process of MTD and MSTD. To compare the detection results, we consider the resulted spike times of each algorithm to calculate the precision and recall. The average precision and recall of MTD and MSTD algorithms for each SNRs are illustrated in Figure 4c,d. The shaded area shows the standard deviation of each precision/recall value at a given SNR. As this figure illustrates, the adaptive detection procedure in MSTD improves the performance of the detection phase in terms of both precision and recall in high SNRs. In low SNRs, however, MSTD shows an improvement over MTD, only in terms of precision. In SNR = 15 dB, MSTD improves the precision and recall of MTD by 6%±0*.*1% and 7%±0*.*1%, respectively. This confirms the effectiveness of our adaptive detection procedure. The proposed adaptive detection consist of two main phases, statistical filtering and multi-point alignment. To examine the contribution of each part in overall detection performance, the precision and recall of applying each part is illustrated in Fig. 4e,f. The base method is achieved by removing both statistical filtering and multi-point alignment from the detection procedure. In terms of precision, the effect of multi-point alignment is higher than the statistical filtering. We believe that the synthesized dataset is not suitable to reveal the effect of statistical filtering, since the synthesized noise is a simple Gaussian noise. Therefore, later in this section, a further experiment with real dataset is done to examine the effect of statistical filtering. In terms of recall, as expected, since the statistical filtering only deals with false alarms, improvement is the result of multi-point alignment.

To further investigate the adaptive detection procedure, we applied it on the top of three detection procedures, including multiresolution Teager energy operator (MTEO)^38^, wavelet-based detection (WBD)^39^, and nonlinear energy operator proposed by Tariq et al.^40^. To compare the results, the precision is compared at a fixed recall rate before and after applying adaptive detection. The results for different SNRs are summarized in Table 1. As can be seen, for both MTEO and NEO, the proposed adaptive detection could improve the detection results. However, for WBD, only in high SNRs a small improvement is observed. We note that the adaptive detection procedure is applied with its default settings and by optimizing such settings for each detection procedure, its results could be probably more improved.

**Table 1:** The effect of the proposed adaptive detection on different spike detection procedures using synthesized dataset.

SNR	MTEO		WBD		NEO	
	B	A	B	A	B	A
1	0.3614	0.3867*	0.5420*	0.5395	–	–
3	0.4079	0.4239*	**0.5795**	0.5786	–	–
5	0.4460	0.4832*	**0.6128**	0.6121	0.0047	**0.0050**
7	0.5282	0.5559*	0.6829*	0.6817	0.2747	0.2918*
9	0.6747	0.6995*	0.8619*	0.8610	0.4198	0.4440*
11	0.8760	0.9082*	0.9796	**0.9797**	0.3206	0.3576*
13	0.9909	**0.9952**	0.9988	0.9989*	0.4551	0.4955*
15	0.9999	0.9999	0.9999	**1.0000**	0.5205	0.5501*

First, different detection procedures are applied on the raw synthesized data with different SNRs. Then, the resulted spike wave-shapes and their corresponding occurrence times are fed to the adaptive detection procedure. Finally, the precision before and after the adaptive detection procedure is calculated at a fixed recall rate. Here, B and A indicate the precision before and after applying adaptive detection, respectively. Significant differences (*p* < 0*.*01) are marked with star. The results indicate an improvement over both MTEO and NEO. However, in WBD, there exists a slight improvement in high SNRs. Thus, the proposed adaptive detection procedure could be applied to different detection procedures to improve the final results. In MTEO method, the threshold parameters of statistical filtering are fitted based on the simulated data.

### Application and validation of the proposed clustering method on synthesized dataset

**Figure 5:**
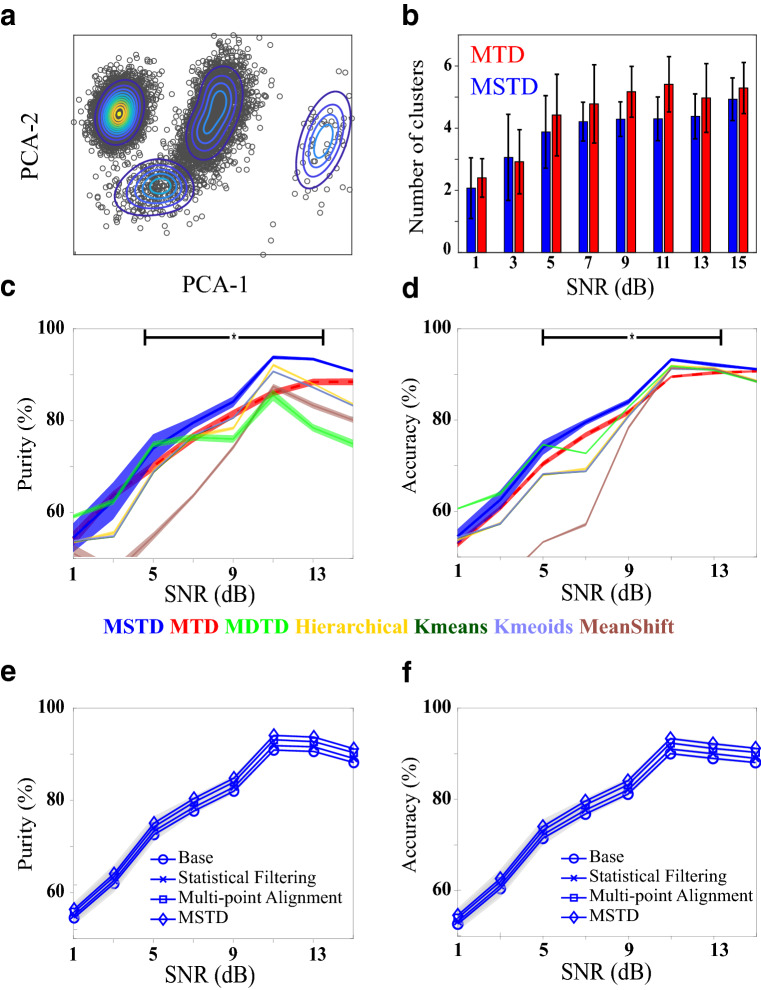
Evaluating clustering phase of the MSTD algorithm using simulated dataset^35,36^ in the clustering phase. (**a**) An example of MSTD fitted on a simulated session. (**b**) The average number of clusters determined by each method for different SNRs. The standard deviation is shown as error bars. (**c**, **d**) The accuracy and purity of clustering outcomes for MSTD compared to six other methods. The shaded area is the standard error. The horizontal line with star shows where the values between MSTD and MTD differ significantly. (**e**, **f**) Investigating the contribution of the proposed multi-point alignment and statistical filtering on clustering performance in terms of purity and accuracy. Results show the higher impact of multi-point alignment than statistical filtering.

After the detection phase, the detected spikes are fed to the clustering methods of MTD and MSTD. For a fair comparison, both methods are applied to the same set of detected spikes in each session with the same SNR. One example session with distribution fitted by MSTD is illustrated in Fig. 5a. The average number of automatically found clusters by each method in different SNRs alongside their standard deviations is demonstrated in Fig. 5b. As can be seen, in a large SNR interval (i.e. 5dB to 13dB), the average number of clusters is close to the real number of clusters (each recording has four neurons) with less standard deviation. Thus, MSTD determines the number of clusters better than MTD. The average accuracy and purity is demonstrated in Fig. 5c,d for MSTD and six commonly used clustering methods including MDTD, hierarchical clustering, Kmeans, Kmedoids, and mean shift clustering. We note that, although methods such as MDTD, hierarchical clustering, Kmeans, and Kmedoids need to be initialized with the correct number of clusters, the accuracy and purity of the proposed method is significantly higher. As shown, in SNRs greater than five, MSTD outperforms other methods in terms of both accuracy and purity. At SNR=11 dB, the accuracy and purity of MSTD are 4%±3% and 7%±4% greater than that of MTD, respectively. As a consequence, the MSTD algorithm better clusters the detected spikes. The contribution of the proposed statistical filtering and multi-point alignment on the overall performance of MSTD is depicted in 5(e,f). In both metrics, the performance of the proposed multi-point alignment is higher than the statistical filtering and is close to the total performance of MSTD. As for precision in detection, again we believe that synthesized dataset is not suitable for evaluation of statistical filtering.

### Application and validation of MSTD on real dataset with ground truth

**Figure 6:**
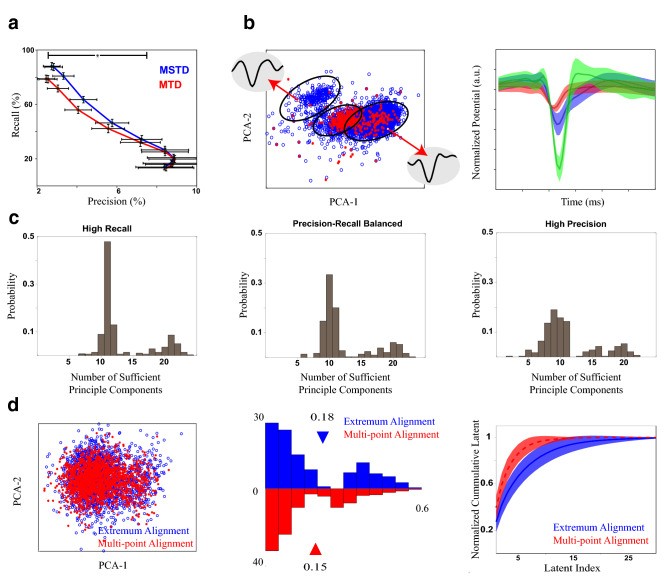
Comparison of MTD and MSTD using the real dataset of simultaneous intracellular and extracellular recordings of rat hippocampus^37,49^. (**a**) The precision-recall curve of the detection results. To achieve this curve, the detection threshold is varied proportional to the noise power. The standard deviation of the precision and recall are indicated using horizontal and vertical error bars, respectively. As can be seen, the precision values are small. The horizontal line with the star shows where the values of both recall and precision differ significantly. (**b**) It shows the reason for small values of precision. A recording sample is visualized using the two first principal components (left). The blue circles show the detected samples, and the red stars show the spikes detected using the ground truth. On the right, the average waveforms of neurons of this sample are illustrated. The clusters are formed using simple manual clustering. The shaded area states the standard deviation. (**c**) The histogram of the number of principal components is needed to describe the spike waveforms, *n*_*c*_, calculated by Eq. (5), for three situations: high recall (left), which is the point with the most recall value in (**a**), precision-recall balanced (middle), which is the point in the middle of high recall and high precision points in (**a**), and high precision (right), which is the point with the most precision value in (**a**). (**d**) The effect of alignment on the cluster compactness. A sample of ground truth spikes of two recordings is visualized using the first two principal components (left). The blue circles and red stars show data for two alignment methods. One is the alignment based on waveform extremum (extremum alignment), and the other is the proposed multi-point alignment. The histogram of within distance of ground truth spikes for extremum alignment and the proposed multi-point alignment (middle), and normalized cumulative latent [the left side of Eq. (5)] is shown for both alignment methods (right). The shaded area is the standard deviation.

In the next experiment, the MSTD and MTD algorithms are examined using a real dataset with ground truth for the time of spikes (rat hippocampus). The extracellular recordings are fed to the detection procedures of MTD and MSTD algorithms. Here, a precision-recall plot is employed to perform a comparison between two detection procedures for a range of precision and recall values, simultaneously. In this way, two procedures could be compared in low and high precision or recall states. To achieve this, the threshold is selected as a variable coefficient of the estimated noise power, i.e. *t*_*s*_ = *c*_*t*_ ×*σ*_*n*_ where *c*_*t*_ ∈ {1*,*1*.*5*,*2*,*2*.*5*,*3*,*3*.*5*,*4*,*4*.*5*,*5}. The result is depicted in Fig. 6a. The standard deviation for each average value of precision and recall is also indicated using horizontal and vertical error bars, respectively. As can be seen, MSTD reaches a higher precision for the same value of recall and vice versa. This improvement is the result of our detection and preprocessing procedures. The reason of the low precision values in Fig. 6a is illustrated in Fig. 6b. As indicated, the ground truth achieved by intracellular recording only contains the spikes of one neuron; however, in a recording, there may exist several neurons which explains the low values for precision. In this example, our manual sorting showed three kinds of neurons in this recording.

Followed by spike detection and then feature extraction, the number of features (*n*_*c*_) needs to be selected using Eq. (5). Here, using a real dataset, we showed the reason of choosing a variable number of features. The distribution for the number of features (i.e. *n*_*c*_) with three different detection thresholds is shown in Fig 6c. The results are shown for three different situations: (i) high recall (*c*_*t*_ = 1), (ii) precision-recall balanced (*c*_*t*_ = 3), and (iii) high precision (*c*_*t*_ = 4*.*5). As seen, in all situations *n*_*c*_ varies in a wide range, demonstrating the variability of spike waveform changes from one recording to another; thus, an adaptive method to select the number of components is required.

Next, we examined the effect of the multi-point alignment procedure on the spike waveforms. The spike waveforms are detected using the provided ground truth times; then two kinds of alignments are applied. The first one is to align waveform according to their minimums (extremum alignment), and the second one is the proposed multi-point procedure. Two samples of neuron spikes are indicated in the left side of Fig. 6d. The alignment procedure seems to make the neuron spikes more compact. To evaluate this, the distribution of within distance of clusters are depicted in the middle plot of Fig. 6d. The alignment procedure significantly (*p* = 5 × 10^−27^) reduced the within distance of the clusters, which means it makes the waveforms of one neuron more similar. Nevertheless, since the intracellular recording provided the ground truth for one neuron, we cannot examine the effect of alignment on between distances of clusters. Another way to examine this effect is the contribution of each principal component in the total variance. The right side of Fig. 6d shows the ratio of explained variance described in Eq. (5) for the two alignment procedures. This figure shows that the proposed multi-point alignment makes the waveforms to concentrate their variances in lower dimensions, which means that spikes could be better described in the lower dimensions.

### Application and validation of MSTD on real dataset without ground truth

**Figure 7:**
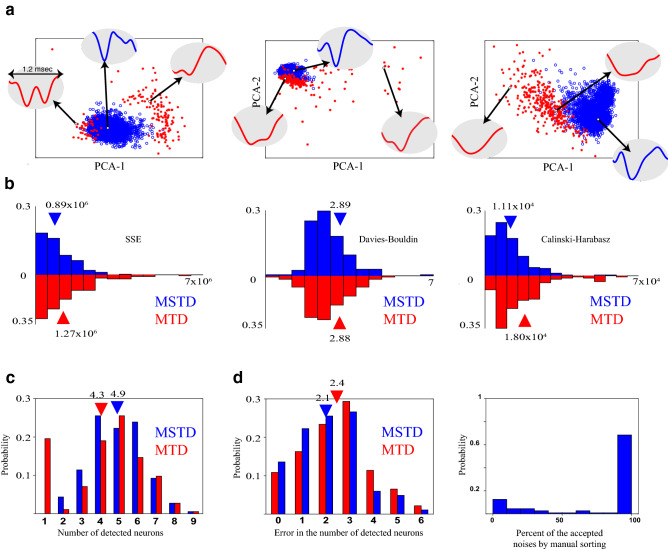
Evaluation of MSTD and MTD using neural recording sessions of the third dataset. (**a**) Three samples of recording sessions. The waveforms that are detected as noise by statistical filtering are illustrated by red stars and the other waveforms by blue circles. (**b**) Investigating the quality of clustering using three blind metrics that do not need any ground truth. In the SSE criterion (left), MSTD outperforms MTD, while in Davies-Bouldin criterion (middle) there exists no significant difference; however, in terms of Calinski-Harabasz criterion, MTD is better. (**c**) The histogram of the number of clusters determined automatically by each method. (**d**) Comparison of automatic spike sorting method with the manual one. The absolute difference between the number of clusters determined automatically by MTD or MSTD and manual sorting (left). The histogram of the percent of the waveforms that are considered as noise both by statistical filtering and manual sorting (right).

Here, we examined the MSTD algorithm with our dataset which had no ground truth. To evaluate the effect of the introduced statistical filtering procedure, three recording samples are shown in Figure 7a. The red stars are detected noises using statistical filtering. Samples of waveforms detected as noise are also indicated. In the PCA space, the detected waveforms of noise are sparsely located away from the concentrated location of spikes. Their waveforms are also highly different from other spikes stating the effectiveness of the proposed statistical filtering procedure. Since there exists no ground truth for this dataset, we used three metrics to evaluate the clustering quality: (i) sum of square errors (SSE), (ii) Calinski–Harabasz criterion^41^, and (iii) Davies–Bouldin criterion^42^. As shown in Figure 7b, the clustering quality of MSTD is significantly better than MTD in terms of SSE (*p* = 5×10^−25^) and is significantly worse than MTD in terms of Calinski-Harabasz criterion (*p* = 10^−6^). Also, there exists no significant difference between the two methods from the Davies-Bouldin criterion point of view (*p* = 0*.*44). Thus, these blind criteria could not actually show significant superiority simultaneously. The distribution of the number of clusters determined by both algorithms are plotted in Figure 7c. It can be inferred that, MSTD tends to have more clusters in this dataset. Also, MTD results in only one cluster in about twenty percent of the recordings.

Finally, to have a better comparison, the resulted clusters of MTD for all sessions have been manually modified by a human operator to achieve manual sorting outcomes^22^. The available operations are merging, deleting, and resorting the clusters. Also, the results of the statistical filtering, i.e. the waveforms which are detected as noise, are provided for a human operator. The operator decides based on the waveform shapes and PCA features of the clusters. Figure 7d shows the absolute difference between the number of clusters determined by the manual sorting and the MTD or MSTD algorithms. The number of clusters in the MSTD is closer to what is achieved by the manual sorting. We also checked the percent of the samples that are detected as noise by statistical filtering that are removed by the operator in the manual sorting process. Results confirm that in approximately 70% of the sessions, more than 90% of samples detected as noise by our statistical filtering procedure are also considered as noise in the manual sorting process.

To sum up the main finding of these experiments, a comparison between the MSTD and MTD algorithms is summarized in Table 2. The algorithms are compared regarding the phases where our main findings are bolded, i.e., adaptive detection and clustering, using two datasets: I) synthesized, and II) a real dataset with ground truth. Considering the adaptive detection phase, four metrics are used. First, the cluster within distance, reduced by 16%. Second, compared to MTD, the number of selected principal components in the feature extraction procedure (see Eq. 5) is decreased by 35%. Finally, as a result, the precision and recall are improved by 14% and 17%, respectively, in the real dataset with the ground truth. Considering the clustering phase, the purity and accuracy are improved by 7% and 4% respectively, in the synthesized dataset.

**Table 2:** Summary of the superiority of the MSTD algorithm over MTD.

Dataset	Adaptive detection				Clustering	
	RCW* (%)	FDR** (%)	Precision (%)	Recall (%)	Purity (%)	Accuracy (%)
D1	–	–	6 ± 0*.*1	7 ± 0*.*1	7 ± 4*.*0	4 ± 3*.*1
D2	16 ± 0*.*1	35 ± 0*.*6	14 ± 2*.*8	17 ± 3*.*4	–	–

All values in the table are the percent of the improvement (decrease or increase) in the metrics. In the table, D1 and D2 refer to the synthesized and real dataset with ground truth. There exist four metrics that show the effectiveness of our adaptive detection procedure: (I) *RCW* Reduction in the clusters within distance (see Fig. 6d), (II) *FDR* Reduction in the number of principal components selected as feature (see Fig. 6d), (III) *Precision* Increase in the precision value (see Figs. 4c,d, 6a), and (IV) *Recall* Increase in recall value (see Figs. 4c,d, 6a). In the clustering phase, there exist two metrics: (I) *Purity* Increase in the resulted clusters’ purity (see Fig. 5c), and (II) *Accuracy* Increase in the accuracy value of the final results (see Fig. 5c). The standard errors of all values are also presented in the table.

*Reduction in the cluster within distance.

**Feature dimension reduction.

## Discussion

In this paper, a novel robust spike sorting algorithm is introduced. In the proposed algorithm, after initial detection in the preprocessing phase, an adaptive detection step is considered to increase the quality of detected spikes. Then, a mixture of skew-t distributions is used for clustering. By evaluating the MSTD algorithm over synthesized and two real datasets, with and without ground truth information, the higher quality of spike sorting and the detection even in noisy channels have been achieved.

Although estimating the skewness parameters adds additional computation complexity to the proposed method, the proposed method has three main contributions: (i) considering non-symmetrical clusters, (ii) new multi-point alignment algorithm, and (iii) statistical filtering for noise removal. It is observed that the proposed method gives several advantages in both detection and clustering phases. In the detection phase, the correct detection rate is increased while the false alarm rate is decreased. Thus, the MSTD algorithm detects more spikes and fewer noises. The spikes detected as noises in our algorithm are also rejected by a human operator in the most cases of manual sorting. Hence, the proposed algorithm decreases the effect of noisy samples, which increases the variability of data and may cause the automatic sorting algorithms to misclassify them within a neuron or as a new one. Applying suggested multi-point alignment compacts the cluster of the spike in the represented space. The lower within-distance clusters increase the quality of spike sorting. The great decrease of error and improvement of clustering purity confirms the performance of skew-t distributions for spike sorting methods. Moreover, the number of detected neurons is significantly closer to the real number of clusters in simulated and manually sorted real data. Therefore, the proposed automatic sorting algorithm is closer to the ultimate goal of spike sorting (i.e., to distinguish spiking activities of different neurons) compared to available solutions.

The spike sorting algorithms face several challenges, including sophisticated noise^43^, non-Gaussian skewed cells^25–27^, and time overlapped spikes due to the simultaneous activities of several neurons^44^. We addressed the first two challenges, while the overlapped spikes is out of the scope of the proposed algorithm. The first challenge, i.e., sophisticated noise, is addressed using statistical filtering, which removes false alarms based on statistical features of wave-shapes. The second challenge, i.e., the skewed cells, is handled by adding skewness in the clustering phase. There are several sorting methods focused on clustering phases; however, they usually applied simple detection methods^22,23^. For instance, the ISO-SPLIT algorithm^22^ introduced a new clustering, while a simple detection method is employed. Furthermore, the challenge of skewed cells is not investigated so far to the best of our knowledge. The problem of drifting clusters in long recordings examined by Shan et al.^24^. They used a mixture of drifting t-distribution clustering methods to handle this problem; however, they employ a simple energy operator for spike detection. Besides, this algorithm cannot model cells with skewed distribution.

Adding the suggested multi-point alignment and considering skewed cells are poorly addressed by current spike sorting methods. Souza et. al. applied the Gaussian mixture model (which is a symmetric model that cannot model skewed cells) with a variety of features based on PCA and wavelet decomposition alongside a threshold crossing event detection^23^. While PCA is the most common feature extracting method for spike sorting, Caro et al. proposed a new feature extraction method based on the shape, phase, and distribution features of each spike^45^. Different solutions were introduced for addressing the high-density multielectrode arrays challenge^46^. The PCA and the mean shift algorithm are employed for sorting by Hilgen and colleagues^46^. The compressed sensing is another solution for the multielectrode arrays challenge^47^. The problem of overlapped spikes could also be handled by applying some post-processing on the combination of neuron templates^18,44,48^. Therefore, different parts of our suggested algorithm can be applied by current spike sorting methods to increase the quality of detected neurons. Nevertheless, adaptive detection and considering skewness in the clustering increase the complexity of the algorithm. However, using parallelism and employing graphics processing units (GPUs) for implementation could abate the complexity.

Our method sought to fill the gap in the presented spike sorting literature by targeting and introducing a robust adaptive detection algorithm. The challenge of not well-formed clusters is considered by applying a mixture of skew-t distribution in the clustering phase. All in all, the proposed spike sorting algorithm made one step toward robustness against two major challenges: complex noise and non-Gaussian or skewed cells. The implementation of the proposed method is freely available as an open-sourced software. All the mentioned parameters can be modified in the toolbox, which makes it easy to work with any kind of data. The presented toolbox is not merely an offline automatic spike sorting algorithm. It also provides a variety of manual functions like merging, resorting, removing, manual clustering in the PCA domain, etc. to improve the quality of the sorting results. Besides, several visualization functions are also provided to guide the user through the sorting procedure.

## Supplementary Information

Supplementary Information.
