# Supplementary material for: An automatic spike sorting algorithm based on adaptive spike detection and a mixture of skew-t distributions
Source: Sci Rep. 2021 Jul 6;11:13925. doi: 10.1038/s41598-021-93088-w (PMC8260722; doi:10.1038/s41598-021-93088-w)
Supplement: Supplementary file 1 — Supplementary Information. [file 41598_2021_93088_MOESM1_ESM.pdf]

An Automatic Spike Sorting Algorithm Based on Adaptive Spike Detection and a Mixture of Skew-t Distributions

Ramin Toosi[1], Mohammad Ali Akhaee[1,*], and Mohammad-Reza A. Dehaqani[2,3,*]

[1]School of Electrical and Computer Engineering, College of Engineering, University of Tehran, Tehran, Iran.
[2]Cognitive Systems Laboratory, Control and Intelligent Processing Center of Excellence (CIPCE), School of Electrical and Computer Engineering, College of Engineering, University of Tehran, Tehran, Iran.
[3]School of Cognitive Sciences, Institute for Research in Fundamental Sciences, Tehran, Iran.
[*] akhaee@ut.ac.ir, dehaqani@ut.ac.ir

Appendix S1: Zero-Phase Filtering

In this method, first the signal is filtered, and then the time reversed version of the filtered signal would be filtered again. Assuming h is the filter response, in the z-transform domain we have:

$$\begin{aligned} &D(z) = R(z)H(z) \\ &R_F(z) = D(z^{-1})H(z) \end{aligned} \tag{S.1}$$

where R is the z-transform of the raw signal (r). When $|z| = 1$ or $z = e^{jw}$, the output reduces to $R_F(e^{jw})|H(e^{jw})|^2$. Thus, the output has zero-phase distortion.

Appendix S2: Expectation-Maximization for Mixture of Skew-t distribution

In the EM algorithm, it is usual to define a membership variable, $Z_i = (Z_{i1}, ..., Z_{ig})$, where

$$Z_{ij} = \begin{cases} 1, & \text{if } s_i \text{ belongs to the component j} \\ 0, & \text{otherwise} \end{cases} \tag{S.2}$$

The completed log-likelihood function can be written as follows:

$$\begin{aligned} \ell_c(\theta|s,t,u,z) = C - \sum_{i=1}^{N}\sum_{j=1}^{g} Z_{ij} \Big[&\log(\pi_j) - \frac{1}{2}\log(\gamma_j) - \frac{u_i}{2}(s_i - \mu_j - \Delta_j t_i)^T \gamma_j^{-1}(s_i - \mu_j - \Delta_j t_i) \\ &+ \frac{v}{2}\log(\frac{v}{2}) + (\frac{v}{2} - 1)\log(u_i) - \log(\frac{v}{2}) - \frac{v}{2}u_i \Big] \end{aligned} \tag{S.3}$$

where C is a constant, and

$$\delta = \frac{\lambda_j}{\sqrt{1+\lambda_j^T\lambda_j}}, \quad \Delta = \Sigma_j^{\frac{1}{2}}\delta_j, \quad \gamma_j = \Sigma_j - \Delta_j\Delta_j^T, \quad t_i \sim N(\mu + \Delta t_i, u_i^{-1}\gamma_j) \tag{S.4}$$

The E-step includes calculating of $Q(\theta|\theta^{(K)}) = E_{\theta^{(K)}}(\ell_c(\theta|s,t,u,z)|s)$, where the superscript (k) indicates the value of the parameters in the k'th iteration. The EM algorithm which finds the parameters of a mixture of p-dimensional skew-t distributions can be summarized as follows:

E-Step: First, consider the following auxiliary parameters.

$$
\begin{aligned}
M^2(\theta) &= (1+\Delta^T\gamma^{-1}\Delta)^{-1} \\
m(\theta,s) &= M^2(\theta)\Delta^T\gamma^{-1}(s-\mu) \\
A(\theta,s) &= \lambda^T\Sigma^{-\frac{1}{2}}(s-\mu) \\
\beta(\theta,s) &= \frac{2\Gamma\left(\frac{v+p+2}{2}\right)(v+d_\Sigma(s,\mu))^{-1}}{\Gamma\left(\frac{v+p}{2}\right)} \frac{T\left(\sqrt{\frac{v+p+2}{v-d_\Sigma(s,\mu)}}A(\theta,s)|v+p+2\right)}{T\left(\sqrt{\frac{v-p}{v+d_\Sigma(s,\mu)}}A(\theta,s)|v+p\right)} \\
\tau(\theta,s) &= \frac{1}{\left(\sqrt{\frac{v+p}{v+d_\Sigma(s,\mu)}}A(\theta,s)|v+p\right)} \frac{\Gamma\left(\frac{v+p+1}{2}\right)}{\pi^{\frac{1}{2}}\Gamma\left(\frac{1}{2}\right)} \frac{(v+d_\Sigma(s,\mu))^{(v-p)/2}}{(v+d_\Sigma(s,\mu)+A(\theta,s)^2)^{(v-p+1)/2}} \\
\xi(\theta,s) &= \beta(\theta,s)m(\theta,s)+M(\theta)\tau(\theta,s) \\
\omega(\theta,s) &= \beta(\theta,s)(m(\theta,s))^2+(M(\theta))^2+M(\theta)m(\theta,s)\tau(\theta,s)
\end{aligned}
\tag{S.5}
$$

The probability of i'th spike belongs to the j'th component, i.e. p_{ij}, is

$$
p_{ij} = \frac{\pi_j ST_p(s_i|\theta_j)}{\sum_{l=1}^{g}\pi_l ST_p(s_i|\theta_l)}
\tag{S.6}
$$

Given $\theta = \theta^{(k)}$, first we calculate $p_{ij}^{(k)}$, then,

$$
\beta_{ij} = p_{ij}^{(k)}\beta(\theta_j^{(k)},s_i) \quad \xi_{ij} = p_{ij}^{(k)}\xi(\theta_j^{(k)},s_i) \quad \omega_{ij} = p_{ij}^{(k)}\omega(\theta_j^{(k)},s_i)
\tag{S.7}
$$

M-Step: In this step, by maximizing $Q(\theta|\theta^{(k)})$, the update rules for the parameters would be obtained as follows:

$$
\begin{aligned}
\pi_j^{(k+1)} &= n^{-1}\sum_{i=1}^{N} p_{ij}^{(k)} \\
\mu_j^{(k+1)} &= \sum_{i=1}^{N}(\beta_{ij}^{(k)}s_i - \xi_{ij}^{(k)}\Delta_j^{(k)}) / \sum_{i=1}^{N}\beta_{ij}^{(k)} \\
\Delta_j^{(k+1)} &= \left[\sum_{i=1}^{N}\xi_{ij}^{(k)}(s_i-\mu_j^{(k+1)})\right] / \sum_{i=1}^{N}\omega_{ij}^{(k)} \\
\gamma_j^{(k+1)} &= \left(\sum_{i=1}^{N}p_{ij}\right)^{-1}\sum_{i=1}^{N}\Big(\beta_{ij}^{(k)}(s_i-\mu_j^{(k+1)})(s_i-\mu_j^{(k+1)})^T \\
&\quad - \left[(s_i-\mu_j^{(k+1)})(\Delta_j^{(k+1)})^T+(\Delta_j^{(k+1)})(s_i-\mu_j^{(k+1)})^T\right]\xi_{ij}^{(k)}+\Delta_j^{(k+1)}(\Delta_j^{(k+1)})^T\omega_{ij}^{(k)}\Big) \\
v^{(k+1)} &= \arg\max_{v}\sum_{i=1}^{N}\log\left(\sum_{j=1}^{g}\pi_j ST_p(s_i|\mu_j^{(k+1)},\Sigma_j^{(k+1)},\lambda_j^{(k+1)},v^{(k)})\right)
\end{aligned}
\tag{S.8}
$$

The update procedure is based on Δ, and γ instead of λ and Σ. These parameters could be recovered as follows:

$$
\lambda_j = \frac{(\gamma_j+\Delta_j\Delta_j^T)^{-\frac{1}{2}}\Delta_j}{\left[1-\Delta_j^T(\gamma_j+\Delta_j\Delta_j^T)^{-1}\Delta_j\right]^{\frac{1}{2}}} \qquad \Sigma_j = \gamma_j+\Delta_j\Delta_j^T
\tag{S.9}
$$

Appendix S3: Detection result comparison with Mountain sort

Detection performance of MSTD and Mountain sort (MS) methods are compared in terms of precision and recall as mentioned in the manuscript. Results are depicted in Figure S.1. We used two versions of the MS method: i) with default settings (MSD) ii) settings that lead to the best precision and recall (MSB). The proposed adaptive detection method in MSTD outperforms MS method in terms of precision and recall. Only MSB in SNR=5dB has better precision than the proposed method.

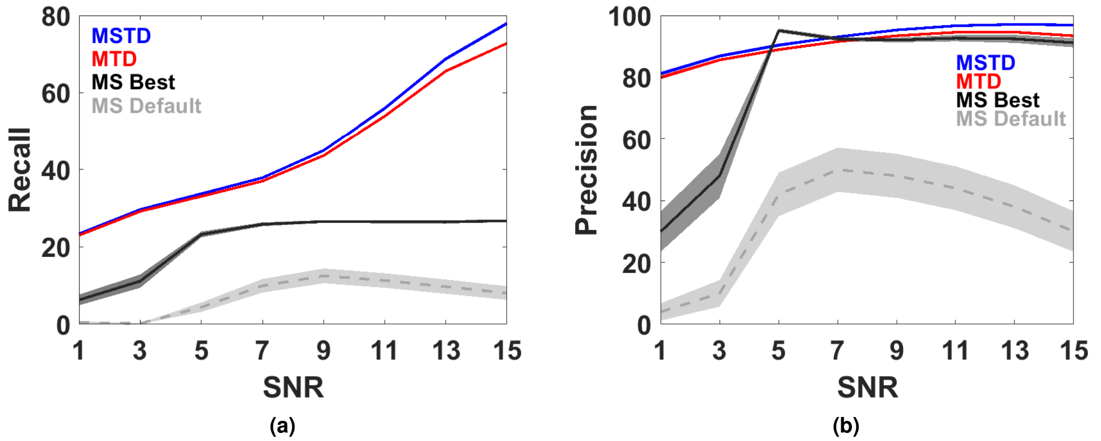

Figure S.1. Comparison of detection performance between the MSTD and Mountain sort methods in terms of precision and recall. (a) Recall of both methods vs SNR. MS method shows a saturation in recall for high SNRs. (b) Precision of both methods vs SNR. MS shows a decrease in precision after SNR=7dB.

Appendix S4: Investigation of the proposed sorting index

The schematic of our proposed spike sorting index (SSI) is illustrated in Figure S.2. There exist three main factors in a fully automatic spike sorting method: i) false alarm, ii) miss detection, ii) clustering error. All these factors are considered in SSI. False alarms are added to the ground truth with a unique cluster index. Miss detections are distributed randomly among the existing clusters. Finally, the clustering error is evaluated using normalized mutual information (NMI). In terms of false alarm, the best SSI would be obtained when the sorting algorithm assigns a unique cluster index to the false alarms. Thus, they could be removed later with more investigation on the resulted neurons (manually or automatically). On the other hand, if the algorithm assigns false alarms to various clusters, the NMI would be reduced. Assigning miss detection randomly to various clusters brings the error distribution on the clusters closer to the uniform one, and consequently reduces the NMI. Therefore, the fewer miss detections, the higher of the SSI. Finally, clustering errors (assigning a wave-shape to the wrong neuron) reduces the NMI between ground truth and predicted clusters. In consequence, all factors affect the proposed SSI. To illustrate the effect of each factor in the SSI, a controlled experiment is designed. In this experiment, the values of two factors were fixed, while the third one ranges between two extremes. Results are depicted in Figure S.3. As can be seen, when miss detection or clustering error increases from zero to one, SSI reduces from one to zero. Also, when the number of false alarms is equal to the number of truly detected spikes, SSI is 0.5.

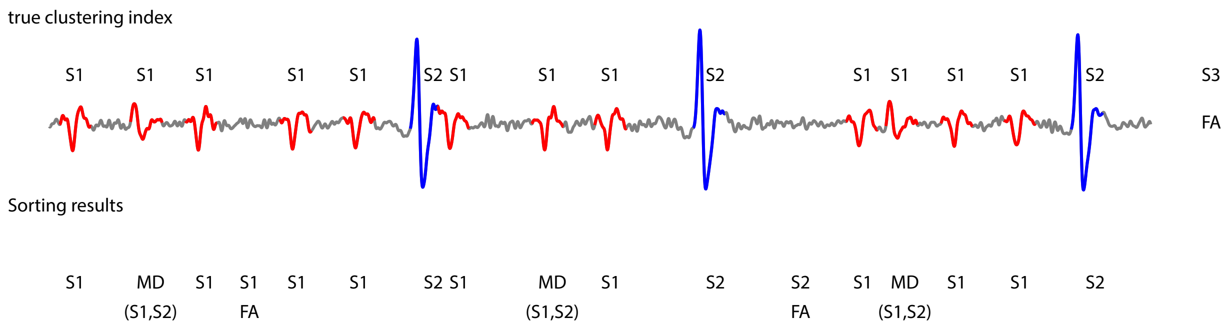

Figure S.2. Illustration of the proposed SSI metric for spike sorting evaluation. There exist two true neurons S1 and S2. The true index for all false alarms (FA) is S3. The predicted index of a miss detection (MD) spike is randomly chosen (S1 or S2). Finally the SSI is the normalized mutual information between the modified predicted indices and true ones.

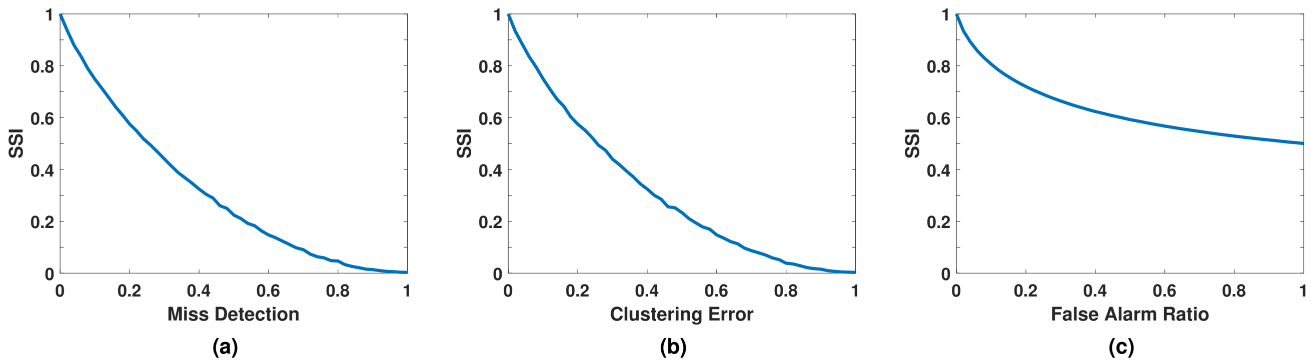

Figure S.3. The effect of miss detection, clustering error, and false alarm on the proposed SSI. (a) Effect of the miss detection rate on SSI. (b) Effect of the clustering error on SSI. (c) Effect of the false alarm on SSI. The x-axis shows the false alarm ratio, where it is defined as the ratio of the number of false alarms (FP) to the number of true detected spikes (TP). In all experiments, two factors are fixed at zero and the third one increases from zero to one. The effect of the miss detection and clustering error is exactly the same with no superiority to each other.

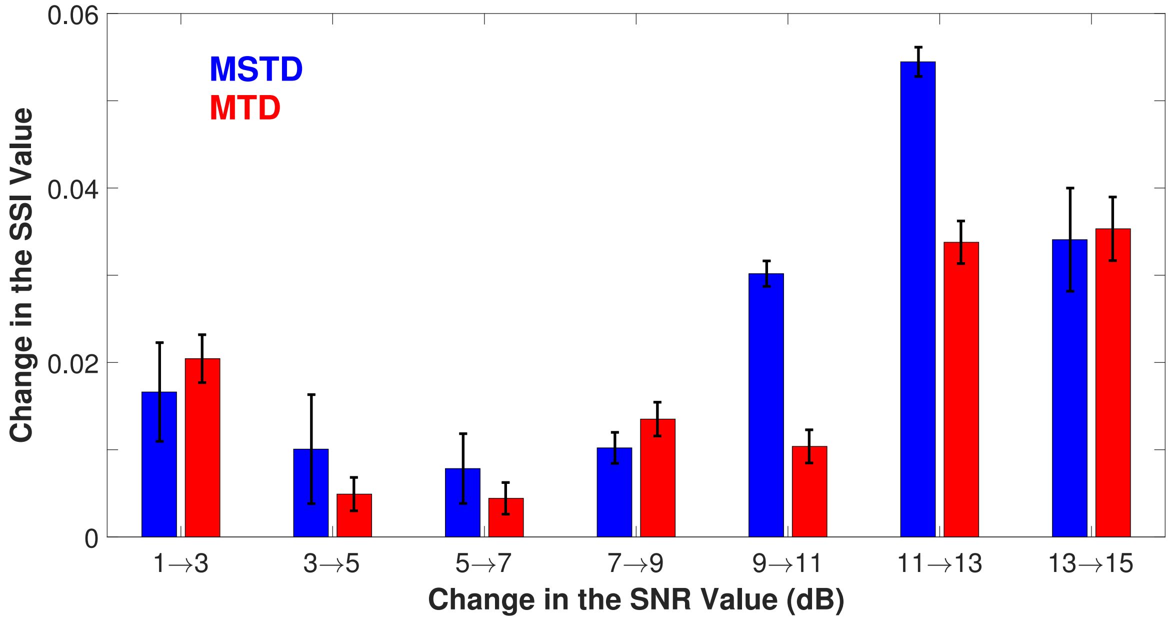

Figure S.4. Comparison of robustness against noise between MSTD and MTD methods. The x-axis shows changes in the SNR value and the y-axis shows changes in the SSI value. The error bars show 95% confidence intervals. Both methods indicate similar robustness except when SNR changes from 9 to 11 dB and 11 to 13 dB.

Appendix S5: Robustness of the proposed method

The amount of the SSI difference between two consecutive SNR values for MSTD and MTD methods is illustrated in Figure S.4. The difference is significant ($pvalue < 0.001$) when SNR changes from 9 to 11 dB and 11 to 13 dB. This experiment shows that the robustness of the MSTD and MTD methods against noise are the same except for two relatively high SNRs, which are less likely to happen in real scenarios.
